# Genome BLAST distance phylogenies inferred from whole plastid and whole mitochondrion genome sequences

**DOI:** 10.1186/1471-2105-7-350

**Published:** 2006-07-19

**Authors:** Alexander F Auch, Stefan R Henz, Barbara R Holland, Markus Göker

**Affiliations:** 1 Center for Bioinformatics (ZBIT), Sand 14, Tübingen, University of Tübingen, Germany; 2 Max Planck Institute for Developmental Biology, Spemannstrasse 37-39, Tübingen, Germany; 3 Allan Wilson Centre for Molecular Ecology and Evolution, Massey University, Palmerston North, New Zealand; 4 Organismic Botany/Mycology, Auf der Morgenstelle 1, Tübingen, University of Tübingen, Germany

## Abstract

**Background:**

Phylogenetic methods which do not rely on multiple sequence alignments are important tools in inferring trees directly from completely sequenced genomes. Here, we extend the recently described Genome BLAST Distance Phylogeny (GBDP) strategy to compute phylogenetic trees from all completely sequenced plastid genomes currently available and from a selection of mitochondrial genomes representing the major eukaryotic lineages. BLASTN, TBLASTX, or combinations of both are used to locate high-scoring segment pairs (HSPs) between two sequences from which pairwise similarities and distances are computed in different ways resulting in a total of 96 GBDP variants. The suitability of these distance formulae for phylogeny reconstruction is directly estimated by computing a recently described measure of "treelikeness", the so-called *δ *value, from the respective distance matrices. Additionally, we compare the trees inferred from these matrices using UPGMA, NJ, BIONJ, FastME, or STC, respectively, with the NCBI taxonomy tree of the taxa under study.

**Results:**

Our results indicate that, at this taxonomic level, plastid genomes are much more valuable for inferring phylogenies than are mitochondrial genomes, and that distances based on breakpoints are of little use. Distances based on the proportion of "matched" HSP length to average genome length were best for tree estimation. Additionally we found that using TBLASTX instead of BLASTN and, particularly, combining TBLASTX and BLASTN leads to a small but significant increase in accuracy. Other factors do not significantly affect the phylogenetic outcome. The BIONJ algorithm results in phylogenies most in accordance with the current NCBI taxonomy, with NJ and FastME performing insignificantly worse, and STC performing as well if applied to high quality distance matrices. *δ *values are found to be a reliable predictor of phylogenetic accuracy.

**Conclusion:**

Using the most treelike distance matrices, as judged by their *δ *values, distance methods are able to recover all major plant lineages, and are more in accordance with Apicomplexa organelles being derived from "green" plastids than from plastids of the "red" type. GBDP-like methods can be used to reliably infer phylogenies from different kinds of genomic data. A framework is established to further develop and improve such methods. *δ *values are a topology-independent tool of general use for the development and assessment of distance methods for phylogenetic inference.

## Background

Molecular phylogenies of many taxonomic groups are based on analyses of single loci. While this approach has led to important insights into the evolution of many groups of interest (consider, as an extreme example, Källersjö et al. [1]), it is also hampered by a number of potential difficulties. For instance, due to effects such as horizontal gene transfer, hybridisation, lineage-sorting, paralogous genes, and pseudogenes, gene trees and species trees do not always agree [2].

Furthermore, length and, hence, information content of individual genes is limited, sometimes causing a lack of resolution in the inferred trees. Saturation is an important problem, in particular if the resolution of relationships between major groups of organisms("deep phylogeny") is aimed at [3]. Nowadays, an increasing number of completely sequenced genomes are available and a growing field of phylogenetic research deals with the question of how to infer reliable phylogenies from this large amount of data to overcome the limitations of single-gene phylogenies.

A relatively obvious approach to phylogenetic analysis of whole genomes is to extract as many genes as possible from the genome sequences, create a multiple sequence alignment from each of the genes and to concatenate all alignments. Datasets in the order of 100, 000 base pairs have been compiled in this way (e.g., [2,4]). Such datasets can be analysed using the same phylogenetic inference tools as single loci datasets.

Difficulties with this approach may arise if orthologous genes cannot be identified with certainty or if the combined sequence length is still too small to give well-resolved trees. Furthermore, the use of concatenated multiple sequence alignments discards information that can be utilised by other methods of phylogenetic inference. For instance, methods that infer trees based on gene content [5-7], gene order [8-10], or content of protein orthologs and folds [11]. When applied to prokaryote phylogeny, these different methodological approaches lead to quite different results [12]. A further loss of information in the concatenated multiple sequence alignment approach may be caused by regions which have to be discarded since they cannot be aligned with certainty [13].

In contrast, a third group of methods does not require to specify genes or orthologs in advance, to create multiple sequence alignments, and to discard unalignable regions, but is able to generate a distance matrix directly from complete genome sequences. Trees can then be inferred using any of the standard distance-based phylogenetic methods (e.g., [14,15]), even though phylogenetic networks [16,17] may be a more powerful way to explore such distance data. Some of these approaches use differences in word-count frequencies [18], complexity-based measures [19] or breakpoint analysis [20] to derive pairwise distance functions.

The methods of particular interest to us in this paper [21-23] rely on identification of local regions of high sequence similarity between two genomes, this is usually done with the popular tool BLAST [24]. Henz et al. [23] recently described the "Genome BLAST Distance Phylogeny" (GBDP) approach and applied it to deep prokaryote phylogeny. In brief, GBDP works by finding a set of high-scoring segment pairs (HSPs) between each pair of genomes, deriving a distance function from these sets, and building a tree or a network using algorithms like UPGMA [25], NJ [26,27], BIONJ [28] or Neighbor-net [16].

Statistical support of individual branches within trees inferred from multiple sequence alignments is usually assessed by bootstrapping [29], which assumes a number of statistically independent individual characters. Similar to some other less commonly used but valuable (and, hence, perhaps underused) phylogenetic methods such as elision [13,30], direct optimisation [31], fixed-states and search-based optimisation [32,33], or pair-wise distances between unaligned sequences from single loci [34-36], the above-mentioned genome distance methods cannot readily be combined with the bootstrap since the whole genome is treated as a single character.

In our view, this potential disadvantage is outweighed by the fact that distance methods may be combined with phylogenetic network techniques, which have some distinct advantages over bootstrapping (e.g., [16,17,37,38]). For instance, bootstrapping cannot distinguish between conflicting signal and low amount of signal, and bootstrapping cannot identify "rogue taxa" (e.g., [39,40]). Furthermore, many evolutionary processes are better represented by networks than by trees [17,37,38,41,42]. Network techniques are better suited than bootstrapping to detect systematic error in phylogenetic analyses, particularly in very large datasets such as genomescale data [17]. Neighbor-net is also much faster than even Neighbor-joining bootstrapping [16]. Since distance methods such as GBDP may also directly use complete genome sequences, their combination with network techniques may be more efficient than bootstrapping of concatenated multiple sequence alignments.

The present article builds on the work of Henz et al. [23] and extends it in several ways. Here, we apply GBDP to completely sequenced plastid and mitochondrion genomes to infer relationships of major eukaryotic groups. Plastid and mitochondrion genomes are highly, sometimes extremely, reduced, and are subject to evolutionary conditions quite different from prokaryote genomes. We were thus interested in whether GBDP would perform as well as with genomes of prokaryotes [23], and if so, under which conditions. Completely sequenced plastid genomes have been used in a number of articles (e.g., [43-47]) to infer phylogenetic relationships based on sequence alignments of many concatenated genes, enabling us to directly compare the GBDP results with respect to, e.g., recovery and placement of major eukaryotic groups and location of primary and secondary endosymbiosis events.

We also examine additional modifications of GBDP. A new distance function based on sequence identity within HSPs is introduced. Different formulae for creating symmetric similarity scores from the asymmetric results of BLAST comparisons are examined, as well as two different formulae to derive distances from similarity values. We also investigate the use of protein-protein BLAST (WUTBLASTX [24]) instead of nucleotide-nucleotide BLAST (NCBI-BLASTN [48]) and two ways of combining the two methods of HSP search. Accuracy of trees inferred from GBDP distances by three well-known (UPGMA, NJ, and BIONJ) and two recently described reconstruction methods (STC [49] and FastME [50]) is measured by comparison with current NCBI taxonomy based on c-scores [23]. The c-score is defined as the number of non-trivial splits in the phylogenetic tree under study which are compatible [51] to the reference topology divided by the total number of non-trivial splits in the test tree. These compatible splits are either already included in the reference topology, or a refinement of the topology, but do not conflict with it. The c-score's denominator is useful to correct for, e.g., a different number of taxa or a different amount of resolution in the test trees. The main factors increasing or decreasing GBDP accuracy were determined by multiple regression analysis with c-score as dependent variable.

Holland et al. [52] described a statistical geometry approach to estimate the departure of a distance matrix from the additivity condition [53], i.e., the degree to which it is not treelike, by computing so-called *δ *values for all quartets of taxa. A similar approach is the Q criterion of Guindon and Gascuel [54], which is also computed from taxon quartets and can be used to assess the treelikeness of a distance matrix. As most distance methods are guaranteed to infer the correct tree from completely additive distances, distance matrices with the least departure from additivity should be preferable [14,52]. An additional advantage of *δ *values is that they are, in contrast to, e.g., c-scores, independent of any preconceived hypothesis on how the true phylogeny looks like. We thus examined quality of each GBDP distance matrix in phylogeny reconstruction directly by measuring its mean *δ *value. As an empirical investigation of the approach described by Holland et al. [52], suitability of *δ *values in predicting phylogenetic accuracy could then be assessed by regression analyses.

## Methods

### Taxon selection

Completely sequenced plastid and mitochondrial genomes were downloaded from NCBI [55] and EMBL [56]. If more than one plastid or mitochondrial genome of the same species was available, we checked them for length differences and randomly selected one sequence representing each of the length classes found. The most recently published completely sequenced plastid genomes that could be considered were *Acorus calamus *[46] and *Pseudendoclonium akinetum *[57]. We also included two completely sequenced genomes of a special kind of organelle found in Apicomplexa as these "Apicoplasts" have previously been shown to be most likely derived from plastids [58]. As outgroup specimens, we included three Cyanobacteria genomes (*Synechococcus *sp., *Synechocystis *sp., and *Thermosynechococcus elongatus*) in the dataset, resulting in a total of 50 genomes for the plastid analyses.

To infer the position of the root in the analyses of mitochondrial genomes, members of the *α*-Proteobacteria genera *Rickettsia *and *Wolbachia *were included in the dataset. Partly due to the lack of plastids in most eukaryotes and partly due to the importance of mitochondrial genes in phylogeny reconstruction in Metazoa, particularly Vertebrates (e.g., [59]), many more completely sequenced genomes are available for mitochondria than for plastids. We thus decided to represent the main lineages within Metazoa-Coelomata, e.g., Arthropoda and Vertebrata, by only a single taxon, respectively, and arrived at a total of 125 mitochondrial (and outgroup) sequences, which we believe to be representative. Including more mitochondrial genomes in the study would have made all analyses considerably more time-consuming and would have made the plastid and mitochondrial data less comparable since mitochondrial genome availability is currently severely biased towards certain Metazoan lineages. Our taxon selection does not imply that the excluded mitochondrial sequences, or the application of the methods described here to these sequences, are devoid of scientific interest. Rather, the related questions are beyond the scope of the present article.

### Variants of genome BLAST distance

The first step in computing any of the GBDP methods explored in the present paper is an all-against-all pairwise comparison of all genomes using BLAST [24,48]. A list of high-scoring segment pairs (HSPs) is determined for each pair of genomes *X *and *Y *including data on location, length, and significance (indicated by an E-value and/or a score) of the individual HSPs. Henz et al. [23] observed that thereafter it is advantageous to determine a maximum subset of HSPs which are non-overlapping in both sequences *X *and *Y*, and that this can be accomplished using the greedy-with-trimming approach. This approach is described fully in [23], in brief, HSPs are selected in decreasing order of length, all of the HSPs that have yet to be selected are trimmed of any overlap with the currently selected HSP and placed back into the sorted list of HSPs still to be selected. Next genome similarity values are inferred from the lists of non-overlapping HSPs, this can be done in different ways.

One method relies on the concept of breakpoints [8-10].

**Figure 1:**
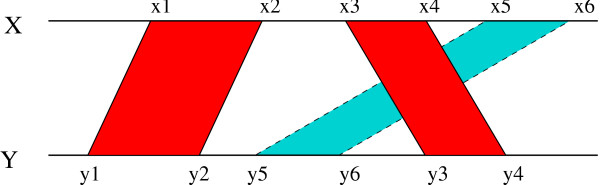
**Identification of Breakpoints**. From a list of high-scoring segment pairs (HSPs) obtained by use of BLASTN or TBLASTX and reduced to a non-overlapping subset by greedy-with-trimming [23], the number of breakpoints can be inferred as follows. In our example, the HSP (*x*5, *x*6, *y*5, *y*6) is located between the HSPs (*x*1, *x*2, *y*1, *y*2) and (*x*3, *x*4, *y*3, *y*4) in genome *Y *but not in genome *X*. This will be counted as a single breakpoint.

In short, a breakpoint occurs if a third, intervening HSP is found between two HSPs in *X*, but not between the two corresponding HSPs in *Y *(see Figure 1 as well as [23] for further details). Let *B*_*X *_and *B*_*Y *_be the number of breakpoints in *X *or *Y*, respectively, and *M*_*X *_and *M*_*Y *_denote the number of matched intervals (i.e., pairs of adjacent HSPs) on the *X *genome or the *Y *genome, respectively. We then define a breakpoint similarity function between *X *and *Y *as

sbreakpoint(X,Y):=1−BX+BYMX+MY     (1)
 MathType@MTEF@5@5@+=feaafiart1ev1aaatCvAUfKttLearuWrP9MDH5MBPbIqV92AaeXatLxBI9gBaebbnrfifHhDYfgasaacH8akY=wiFfYdH8Gipec8Eeeu0xXdbba9frFj0=OqFfea0dXdd9vqai=hGuQ8kuc9pgc9s8qqaq=dirpe0xb9q8qiLsFr0=vr0=vr0dc8meaabaqaciaacaGaaeqabaqabeGadaaakeaacqWGZbWCdaWgaaWcbaGaemOyaiMaemOCaiNaemyzauMaemyyaeMaem4AaSMaemiCaaNaem4Ba8gcbiGae8xAaKMae8NBa4Mae8hDaqhabeaakiabcIcaOiabdIfayjabcYcaSiabdMfazjabcMcaPiabcQda6iabg2da9iabigdaXiabgkHiTmaalaaabaGaemOqai0aaSbaaSqaaiabdIfaybqabaGccqGHRaWkcqWGcbGqdaWgaaWcbaGaemywaKfabeaaaOqaaiabd2eannaaBaaaleaacqWGybawaeqaaOGaey4kaSIaemyta00aaSbaaSqaaiabdMfazbqabaaaaOGaaCzcaiaaxMaadaqadiqaaiabigdaXaGaayjkaiaawMcaaaaa@54A0@

A distance equivalent of this formula was presented by Henz et al. (see [23], equation (4)).

An entirely different approach is based on the proportion of nucleotides (or amino acids if TBLASTX is used) found in the set of non-overlapping HSPs compared to the total number of nucleotides, i.e., the length of the genome. Let |*X*_*match*_| and |*Y*_*match*_| be the number of base pairs covered by the selected non-overlapping HSPs in *X *or *Y*, respectively, and |*X*| and |*Y*| be the total length of the respective genome. Similarity formulae may then be defined as follows:

smatch(X,Y):=|Xmatch|+|Ymatch||X|+|Y|     (2)
 MathType@MTEF@5@5@+=feaafiart1ev1aaatCvAUfKttLearuWrP9MDH5MBPbIqV92AaeXatLxBI9gBaebbnrfifHhDYfgasaacH8akY=wiFfYdH8Gipec8Eeeu0xXdbba9frFj0=OqFfea0dXdd9vqai=hGuQ8kuc9pgc9s8qqaq=dirpe0xb9q8qiLsFr0=vr0=vr0dc8meaabaqaciaacaGaaeqabaqabeGadaaakeaacqWGZbWCdaWgaaWcbaGaemyBa0MaemyyaeMaemiDaqNaem4yamMaemiAaGgabeaakiabcIcaOiabdIfayjabcYcaSiabdMfazjabcMcaPiabcQda6iabg2da9maalaaabaWaaqWaceaacqWGybawdaWgaaWcbaGaemyBa0MaemyyaeMaemiDaqNaem4yamMaemiAaGgabeaaaOGaay5bSlaawIa7aiabgUcaRmaaemGabaGaemywaK1aaSbaaSqaaiabd2gaTjabdggaHjabdsha0jabdogaJjabdIgaObqabaaakiaawEa7caGLiWoaaeaadaabdiqaaiabdIfaybGaay5bSlaawIa7aiabgUcaRmaaemGabaGaemywaKfacaGLhWUaayjcSdaaaiaaxMaacaWLjaWaaeWaceaacqaIYaGmaiaawIcacaGLPaaaaaa@6127@

smcorr(X,Y):=|Xmatch|+|Ymatch|2min⁡(|X|,|Y|)     (3)
 MathType@MTEF@5@5@+=feaafiart1ev1aaatCvAUfKttLearuWrP9MDH5MBPbIqV92AaeXatLxBI9gBaebbnrfifHhDYfgasaacH8akY=wiFfYdH8Gipec8Eeeu0xXdbba9frFj0=OqFfea0dXdd9vqai=hGuQ8kuc9pgc9s8qqaq=dirpe0xb9q8qiLsFr0=vr0=vr0dc8meaabaqaciaacaGaaeqabaqabeGadaaakeaacqWGZbWCdaWgaaWcbaGaemyBa0Maem4yamMaem4Ba8MaemOCaiNaemOCaihabeaakiabcIcaOiabdIfayjabcYcaSiabdMfazjabcMcaPiabcQda6iabg2da9maalaaabaWaaqWaceaacqWGybawdaWgaaWcbaGaemyBa0MaemyyaeMaemiDaqNaem4yamMaemiAaGgabeaaaOGaay5bSlaawIa7aiabgUcaRmaaemGabaGaemywaK1aaSbaaSqaaiabd2gaTjabdggaHjabdsha0jabdogaJjabdIgaObqabaaakiaawEa7caGLiWoaaeaacqaIYaGmcyGGTbqBcqGGPbqAcqGGUbGBcqGGOaakdaabdiqaaiabdIfaybGaay5bSlaawIa7aiabcYcaSmaaemGabaGaemywaKfacaGLhWUaayjcSdGaeiykaKcaaiaaxMaacaWLjaWaaeWaceaacqaIZaWmaiaawIcacaGLPaaaaaa@6819@

A distance equivalent of the second formula was presented by Henz et al. (see [23], equation (3)). They observed that it performed better than the equivalent of the first similarity function if some genomes were essentially subsets of other genomes because their evolutionary history included a considerable number of gene losses.

We now introduce a fourth similarity (and, hence, distance) function based on the proportion of identical base pairs within the set of non-overlapping HSPs to the total length of this set. Defining *I *as the sum of the number of identical base pairs over all HSPs, and *H *:= ∑_*h*∈*HSPs *_max(|*X*_*h*_|, |*Y*_*h*_|) as the sum of the lengths of the larger interval for each HSP, we obtain

sid(X,Y):=1H     (4)
 MathType@MTEF@5@5@+=feaafiart1ev1aaatCvAUfKttLearuWrP9MDH5MBPbIqV92AaeXatLxBI9gBaebbnrfifHhDYfgasaacH8akY=wiFfYdH8Gipec8Eeeu0xXdbba9frFj0=OqFfea0dXdd9vqai=hGuQ8kuc9pgc9s8qqaq=dirpe0xb9q8qiLsFr0=vr0=vr0dc8meaabaqaciaacaGaaeqabaqabeGadaaakeaacqWGZbWCdaWgaaWcbaGaemyAaKMaemizaqgabeaakiabcIcaOiabdIfayjabcYcaSiabdMfazjabcMcaPiabcQda6iabg2da9maalaaabaGaeGymaedabaGaemisaGeaaiaaxMaacaWLjaWaaeWaceaacqaI0aanaiaawIcacaGLPaaaaaa@3DE3@

Again, this function works equivalently with TBLASTX instead of BLASTN, if we replace nucleotides by amino acids.

Literature definitions of the term "similarity" usually agree that similarity values should be constrained between 0 (inclusively) and 1 (inclusively), a condition which holds for the formulae listed above by definition. There is, however, no unique way to define "distance" and no unique formula to derive distance values from similarity values. Let *d*(*X*, *Y*) denote the distance between *X *and *Y *to be computed from the similarity function. The most important options for conversion (e.g., [60,61]) are

*d*(*X*, *Y*) := 1 - *s *(*X*, *Y*)     (5)

and

*d*(*X*, *Y*) := -log(*s*(*X*, *Y*))     (6)

Most formulae described for computing distances from multiple DNA or protein alignments use a logarithmic derivation to correct for saturation effects in the sequence data (e.g., see [62]). Here we apply both formulae to all above-mentioned similarity functions and test their relative perfomance *a posteriori*.

Phylogenetic methods which infer trees from pairwise distance matrices usually expect the distances to be symmetric, i.e., they require that *d*(*X*, *Y*) = *d*(*Y*, *X*) holds even if *X *is not equal to *Y*. BLAST, however, is asymmetric by definition [24]. We therefore inferred symmetric genome BLAST distances in three ways: as the average [23], minimum, or maximum value of *d*(*X*, *Y*) and *d*(*Y*, *X*). We examined whether the quality of the distances and the inferred tree is affected by the choice of approach.

Another way to modify the GBDP approach is to use TBLASTX instead of BLASTN as already proposed by Henz et al. [23], i.e., to search for homologies at the protein level instead of at the nucleotide level. Both BLAST methods could also be combined. As the greedy-with-trimming approach already sorts HSPs by decreasing length, one way of combining BLASTN and TBLASTX HSPs is to sort them together, so that the usually longer HSPs derived from TBLASTX suppress shorter overlapping BLASTN HSPs. A more equally-weighted method of combination is to compute BLASTN and TBLASTX genome distance matrices separately and to determine the average of both matrices afterwards (see [63,64] for other examples of distance matrix averaging). Before inferring the mean matrix, distance matrices usually need to be brought to the same scale. A reliable and generally applicable method of rescaling is the so-called ranging procedure which consists of dividing all values in one matrix by the maximum value observed in that matrix [61,65]. We examined four possibilities for performing HSP search: use of either BLASTN or TBLASTX alone, or a combination of BLASTN and TBLASTX either before or after distance matrix computation.

Note that it has not been established for any of the above-mentioned GBDP methods that they will necessarily result in a distance matrix which is metric, i.e., obeys to the triangle inequality (e.g., [66]). Hence, they cannot be called "distances" in a strictly mathematical sense. Nevertheless, as pointed out by Felsenstein [62], this is also true for phylogenetic distance formulae based on multiple sequence alignments such as the Jukes-Cantor equation, and, more importantly, "most distance methods do not absolutely require the Triangle inequality to hold".

### Quality assessment of distances and phylogenies

The quality of the distance formulae described above for phylogeny reconstruction was assessed in two ways. First, we compared the topology of phylogenetic trees inferred from the respective distance matrix with the topology of the NCBI taxonomy of the taxa involved (NCBI [67]) by computing the c-score. The NCBI taxonomy tree can be regarded as an estimate of the "true" phylogeny, although it is not fully resolved.

Three standard distance-based phylogeny reconstruction methods were applied to the distance matrices, i.e., UPGMA [25] and NJ [26,27] as implemented in the Phylip package version 3.63 [68] as well as BIONJ, a modification of the NJ algorithm described and implemented by Gascuel [28]. Additionally, two more recently described distance methods were considered, FastME [49] and shortest triplet clustering (STC, [50]). Both are said to be faster than any known NJ variant and more accurate, particularly if large numbers of taxa are involved. FastME was run under the balanced minimum evolution criterion [69] including balanced NNI branch swapping.

Compared to trees, phylogenetic networks are more general graphs and may contain conflicting branching patterns. Such networks can be used to visualize hybridisation, horizontal gene transfer, recombination events or noise in the data due to finite length of sequences or inadequacy of the statistical model used to infer pairwise distances [37]. Several methods are available to infer networks from distance data (e.g., [38]). Here, the recently described agglomerative Neighbour-net algorithm [16] as implemented in *SplitsTree4 *[17] was used to visualize treelikeness of and phylogenetic information contained in selected distance matrices. Treelikeness cannot appropriately be visualised by means of tree reconstruction methods such as Neighbor-joining since these will force every distance matrix into a tree irrespective of how treelike it actually is. Neighbor-net is fast and frequently leads to greater resolution than older methods like split decomposition [16]. On the other hand, it would not make much sense to compute c-scores from phylogenetic networks to assess topological accuracy. Since methods such as Neighbor-net are designed to allow the simultaneous representation of multiple trees, or at least to display conflicting signal as well as other non-treelike aspects of the data, the c-score would clearly underestimate topological accuracy [23]. The latter is much better represented by the c-score of a phylogenetic tree, which most likely consists of a subset of the splits represented in a network if inferred from the same distance matrix.

Holland et al. [52] introduced the computation of so-called *δ *values for each quartet of taxa present in the whole dataset (see also [54]). A lower *δ *value indicates greater correspondence to Buneman's condition of tree additivity [53] which is fully satisfied, if *δ *approaches zero. *δ *values can be averaged over individual taxa; in that case, large values of *δ *may, e.g., indicate hybrids [52] or taxa whose evolutionary history, as far as represented by its sequence data at hand, has a particularly low fit to the distance method in use. One can also compute the mean *δ *value of complete distance matrices which indicates suitability of the respective data or the distance function applied to these data for phylogeny reconstruction in general. A script written in the python language was used to compute *δ *values of the individual genomes as well as average *δ *values under all distance approaches considered here. (The script is available on request by emailing b.r.holland@massey.ac.nz)

A complete data set is available for download (in CSV format), containing all reconstructed phylogenetic trees and their corresponding c-scores and *δ *values [see Additional file 1].

### Regression analysis

The individual distance functions described above were recoded into their four independent components (equation (1), (2), (3), or (4); equation (5) or (6); average, minimum, or maximum of asymmetric distance values; BLASTN, TBLASTX, BLASTN after TBLASTX, or matrix combination) each of which was treated as a single qualitative variable with four, two, three, or four possible states, respectively [70]. The phylogenetic reconstruction methods are also easily coded as a single quantitative variable with five states (UPGMA, NJ, BIONJ, FastME, or STC). Two multiple linear regression approaches were used:

1. *δ *value as dependent on the distance components.

2. c-score as dependent on distance components and tree reconstruction methods.

All statistical analyses were carried through using the R package (version 2.1.1, [71]). R automatically recodes qualitative variables into a set of binary variables (e.g., [70]) carrying the same information and suitable for linear regression. R also provides a step-wise regression procedure to eliminate insignificant variables based on the Akaike information criterion (AIC; e.g., [72]). The AIC tries to achieve a balance between model simplicity (the number of parameters used to explain the data) and model likelihood, in accordance with the well-known principle called Ockham's razor (e.g., [73,74]). In each step of the procedure, a variable which, according to the AIC, does not significantly improve the fit of the regression model to the data is removed and all regression parameters such as regression coefficients and t values [75] are recomputed. The step-wise elimination stops when all explanatory variables left make a significant contribution.

## Results and discussion

### Which distance function is optimal?

Table 1 depicts the results of an AIC-based step-wise elimination approach to multiple linear regression performed with R [71]. Besides revealing which distance formulae are most reliable (as discussed in the following paragraphs), regression analysis also indicates that *δ *values work well in predicting phylogenetic accuracy. The *δ *variable is able to represent most of the differences between the distance functions. With data transformed to z-scores, 61.7% of the variance in c-score is explained, if *δ *value alone is used as independent variable. Together with reconstruction methods, *δ *explains 62.1%; *δ*, significant distance parameters (Table 1), and reconstruction methods together explain 87.0% of the variance in c-score.

Henz et al. [23] observed that breakpoint distances performed very poorly in comparing prokaryote genomes which is in accordance with the commonly held view that breakpoint methods lead to reliable results only if the genomes are sufficiently co-linear. Regression analyses indicate that, if applied to plastid and mitochondrion data, breakpoint distances as based on equation (1) had by far the worst performance of all distance formulae with respect to *δ *values and c-scores. We conclude that on the taxonomic level examined here neither mitochondrion nor plastid genomes have a sufficient amount of co-linearity for the breakpoint method to be successful.

It also follows from the regression analysis that results obtained with distances based on nucleotide (or amino acid) identity within HSPs relative to the total length of HSPs (equation (4)) are inferior to those obtained with the matched distance variants (equations (2) and (3)), an observation which could be explained as follows. If two taxa are only distantly related, HSPs will only be found in the most conservative parts of their genomes. It may well be that these loci are so conserved that nucleotides are mostly identical within these HSPs. More closely related taxa, on the other hand, will share more HSPs some of which will correspond to less conserved loci displaying more disagreement between the two sequences. If plotted against corresponding matched distance values (not shown), HSP identity distances at first appear linearly related, but decrease again for largest values of matched distance, supporting our interpretation. It could be argued that the HSP identity distance approach should be used only if genomes are not too distantly related, although in most cases it would still perform much better than breakpoint distances. With mitochondrial genomes, the tied highest c-score was achieved by one of the matched distances and one of the HSP identity distance variants.

Large differences in genome length are often due to a considerable loss of genes related to particular ecological adaptations such as parasitism or mutualism [76]. The main difference between the two matched similarity formulae examined here is that equation (3) attempts to correct for presence of such genomes heavily reduced in size. Interestingly, equation (2) performed better than the corrected one with respect to both *δ *value and c-score (Table 1). On the other hand, formula (3) always led to a correct placement of the reduced genome of *Epifagus virginiana *(not shown; placement similar to Figure 4), whereas *Epifagus *was wrongly located at the base of Angiosperms, if equations (3) and (5) were combined (Figure 5). However, if formula (2) was used in conjunction with (6), the position of *Epifagus *was correctly revealed (Figure 4). We suggest that formulae not corrected for genome size may usually be superior, but that the size-corrected one should be preferred in case of evidence for extreme gene loss in one or more of the genomes investigated [23]. It seems worth noting that use of HSP identity distances also led to *Epifagus *misplacement at the root of Angiosperms (not shown) similar to Figure 5, even though equation (4) does not refer to total genome lengths.

Deriving distances from pairwise similarities as their negative logarithm (equation (6)) performs worse than subtraction from 1 (equation (5)) with respect to *δ *value, but has no significant effect on c-score (Table 1). On the contrary, using the logarithm may improve topological accuracy with respect to taxa with extreme genome modifications like *Epifagus *(compare Figures 4, 5). However, the suggestion of Holland et al. [52] to remove those taxa with largest individual *δ *values would also have been of use with size-uncorrected distances derived by subtraction, as *Epifagus *had a *δ *value of 0.3681 compared to the average *δ *of 0.1629 obtained with the distance matrix underlying Figure 5. We hypothesise that in most cases choosing either subtraction or negative logarithm only has an effect on scaling.

In the multiple sequence alignment setting distances are typically corrected for multiple changes, such corrections require an explicit model of nucleotide substitution [62]. A challenge for future research could be to relate GBDP distance functions to models of genome evolution, so that similar types of correction could be applied. However, it would also be interesting to gather more data on the behaviour of *δ *values when applied to multiple sequence alignments under different types of correction. Possibly, p-distances sometimes result in better *δ *values. It may well be that the most complex model or the best model according to the maximum likelihood criterion does not result in the lowest *δ *values [52].

We did not observe significant differences in performance between the average, maximum, or minimum value approach to conversion of asymmetric into symmetric distance values. Asymmetric GBDP distance values are due to an artefact of using BLAST [24] and have no obvious biological meaning. Furthermore, numeric differences between corresponding asymmetric values were in general quite low, although they increased with increasing absolute value of their average (not shown) and thus could to some degree correspond to the variance of these distances.

### Which HSP search and which reconstruction method is optimal?

Regression analysis (Table 1) indicates that replacing BLASTN by TBLASTX leads to a small improvement in the results and that a combination of TBLASTX and BLASTN at the HSP level (weighting TBLASTX HSPs more heavily than BLASTN HSPs) works even better. Combination at the distance matrix level still performs better than using BLASTN alone, although less significantly so than do TBLASTX and combination at the HSP level. We conclude that this difference is due to greater sequence conservation at the protein level, hence, TBLASTX gives better resolution, especially at the backbone of the phylogenetic trees. It also seems plausible that nucleotide and protein sequences, respectively, may contain different information and that combining both levels in some way is useful in extracting phylogenetic signal from the data.

On the other hand, the effect of the different methods of HSP determination and combination is much less than the effects of either choice of data type (mitochondria vs. plastids), or choice of distance function (breakpoint, matched, or HSP distance). Whereas TBLASTX HSP search takes much longer than BLASTN search, trees inferred from distances based on TBLASTX are not considerably more accurate. Computation time is no severe problem with the mitochondrial and plastid data examined here, but may be limiting if longer sequences like prokaryotic genomes [23] and/or many more taxa (e.g., all mitochondrion genomes currently accessible) are examined. Therefore, the use of BLASTN may be the more efficient method under most conditions.

**Figure 2:**
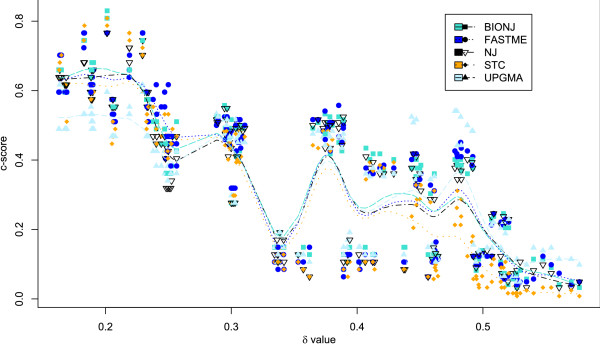
**Comparison of reconstruction methods**. Comparison of distance functions and reconstruction methods. The graph shows how phylogenetic accuracy (c-score) is dependent on distance quality (*δ *value). Each distance matrix is associated with a certain *δ *value, which is lowest in case of best distance quality; each phylogenetic tree computed is associated with a certain c-score, which is highest in case of optimal agreement between the tree and the reference topology. For each distance matrix, trees were inferred using BIONJ (squares), FastME (circles), NJ (open inverted triangles), STC (diamonds), and UPGMA (triangles). To illustrate the behaviour of these individual methods of phylogenetic inference, cubic splines were used; the number of 15 degrees of freedom for the splines apparently optimal to summarize the shape of the data was found by careful optical comparison. For instance, the splines show that UPGMA performs relatively poorly with best distance values, whereas with high *δ *values (i.e., low distance quality) STC performs worst of all tree inference methods examined. See table 1 for a more detailed exploration of the interrelationships of topological accuracy, distance quality, and distance function parameters by multiple linear regression.

With respect to tree reconstruction methods, regression results indicate that BIONJ [28] works best overall, but that the original NJ algorithm [26,27] and the more recently described FastME method [49] do not perform significantly worse. Mean c-score values were 0.3899, 0.3888, 0.3790, 0.3601, and 0.3493 for BIONJ, FastME, NJ, UPGMA, and STC; more importantly, the best values for each reconstruction method were 0.8298, 0.7660, 0.7660, 0.6170, and 0.8085 respectively. Given that FastME has a lower time complexity than BIONJ and NJ [49], we conclude that the former method is preferable with this kind of distances, especially if large numbers of taxa are involved. The low c-scores of the UPGMA trees are as expected, like other clustering methods, UPGMA [25] is known to work well only if the distances at least approximately satisfy the ultrametric condition. This condition usually only holds, if rates of evolutionary change do not vary very much between lineages (e.g., [77]). However, UPGMA performed surprisingly well with low-quality distances (Figure 2).

Considering the results of Vinh and Von Haeseler [50], the relatively poor performance of Shortest Triplet Clustering (STC) is surprising. These authors demonstrated that with large numbers of taxa STC performs better than NJ, BIONJ, and GME or BME (the latter two methods can be used to produce a starting tree in FastME). Vinh and Von Haeseler [50] also showed that these five algorithms work equally well if they are used to initially build a tree which is then improved by NNI branch swapping under the balanced minimum evolution criterion (BNNI [49,69]). Here we only used BNNI in conjunction with FastME, so the performance of STC should have been worse than that of FastME, but better than that of either NJ, BIONJ, and UPGMA. However, an important difference between Vinh and Von Haeseler [50] and the present study is that we included some distance data of apparently very low quality. Figure 2 indeed shows that STC performs particularly poorly for distances with a high *δ *value, but that it works well on high quality distance matrices, in accordance with Vinh and Von Haeseler [50]. Furthermore, these authors focussed on the performance of reconstruction methods with very large (> 1000) numbers of taxa. It must also be emphasised that distance quality, as measured by *δ *values, explains a much larger part of the variance in c-score than does the reconstruction method factor (see above).

### Phylogenetic aspects

Regression results indicate that plastid genomes generally performed significantly better than complete mitochondrial sequences with respect to both *δ *values and c-scores, i.e., correspondence of the resulting trees to current NCBI taxonomy. Indeed, *δ *values of distance matrices ranged from 0.5767 to 0.2882, if inferred from mitochondrial genomes, but from 0.4564 to 0.1629, if inferred from plastid genomes. c-scores ranged from 0.0082 to 0.5574 for mitochondrial data, but from 0.0638 to 0.8298 for plastid data compared to a maximum of 0.727 found by Henz et al. [23] when applying GBDP to 91 prokaryotic genomes. Underlying genome data was one of the most important factors in explaining distance quality (Table 1).

**Figure 3:**
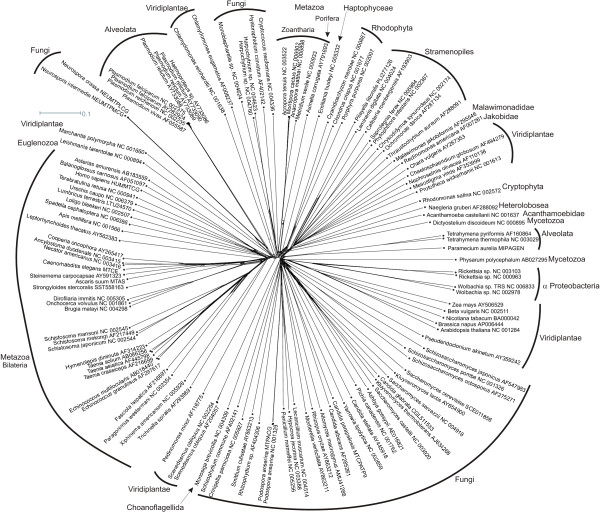
**Neighbor-net reconstruction based on GBDP distances between whole mitochondrion genomes**. The GBDP variant used was derived by scaling and averaging distance matrices based on HSP search with BLASTN and TBLASTX, respectively. Further settings were use of equations (2) and (6) and averaging of asymmetric distance values. With BIONJ as tree reconstruction method, this distance matrix achieved the highest c-score (0.5574), i.e., highest topological accuracy according to the current NCBI taxonomy, of all GBDP trees inferred from mitochondrial genomes. The matrix' *δ *value was 0.2946, indicative of a relatively high distance quality compared to the other results obtained with mitochondrial genomes. Bacterial outgroup taxa and major eukaryotic groups are indicated. Note the apparently low resolution of the network as compared to the best results obtained with plastid genomes (Figs. 4 and 5). Nevertheless, quite a few subgroups of the major eukaryotic groups are well recovered.

We conclude that mitochondrial genomes are saturated for comparisons of the members of the main eukaryotic groups. Maximum values in distance matrices derived from mitochondria were generally larger than those found in corresponding distances derived from plastid genomes (not shown). Consequently, the best mitochondrial phylogenies look rather unresolved (Fig. 3).

Furthermore, some major eukaryotic groups don't appear as monophyletic in the network, although they are frequently split into only a few different clusters. For instance, Metazoa are divided into Porifera (*Axinella*) and Zoantharia (*Acropora *to *Metridium*) on the one hand and Bilateria (*Asterias *to *Trichinella*), i.e., all remaining included Metazoan taxa on the other hand. Fungi are almost completely recovered, except *Cryptococcus*, four Chytridomycete taxa (*Monoblepharella *to *Hyaloraphidium*), and the highly derived *Allomyces*, which are misplaced. Some smaller taxonomic groups of similar rank are completely recovered like *α*-Proteobacteria (outgroup), Rhodophyta and, although less clearly so, stramenopiles. In contrast, Viridiplantae (green algae and land plants; "Chloroplastida" according to [78]) are split into a considerable number of clusters.

However, several subclades of main eukaryotic groups are recovered. For instance, Platyhelminthes (*Schistosoma *to *Paragonimus*) and Coelomata (*Asterias *to *Apis*) within Metazoa as well as Embryophyta (*Arabidopsis *to *Zea*) within Viridiplantae appear as clusters in the phylogenetic network. We suggest that mitochondrial data are of greater use when applied within the major groups of eukaryotes, e.g., within Deuterostomia, and that it would be interesting to apply GBDP to such taxon sets. Other authors were indeed able to construct robust phylogenies from unaligned mitochondrial genomes within, e.g., Vertebrates [19].

On the other hand, we see no evidence that the low backbone resolution of our mitochondrial network is due to shortcomings of the GBDP methodology. To our knowledge, mitochondrial genome-scale analyses through concatenation of single loci and inference of trees using standard methods have not been conducted so far with a taxon sampling as comprehensive as ours. This may even be impossible, since mitochondrial genomes of diverse eukaryotic groups may not share a sufficient number of loci. An advantage of GBDP-like methods (if combined with phylogenetic network techniques) over standard methods based on multiple sequence alignment is that the former do not assume that homology of single nucleotides or amino acids can be established throughout the loci. Here, we were able to compute distance matrices and to assess resolution from the phylogenetic networks without any necessity to preprocess the genomic data.

In contrast to mitochondrial genomes, best plastid-based GBDP distances and trees were of high quality as measured by *δ *value as well as c-score, and are worth discussing in greater detail. A possible limitation of the c-score in assessing quality of phylogenetic trees derived from plastid genes or genomes is that the reference taxonomy, as far as it relies on molecular data, is usually based on nuclear genes. Whereas it is now generally assumed that primary endosymbiosis giving rise to plant-like eukaryotes occurred only once [43-45,78], events like secondary or even tertiary endosymbiosis should lead to incongruence between plastid-derived and nucleus-derived molecular phylogenies. However, we observed that splits potentially causing such conflict (e.g., a group comprised of all plant-like eukaryotes possessing primary plastids surrounded by two membranes) are not included in current NCBI taxonomy, which is far from being completely resolved.

**Figure 4:**
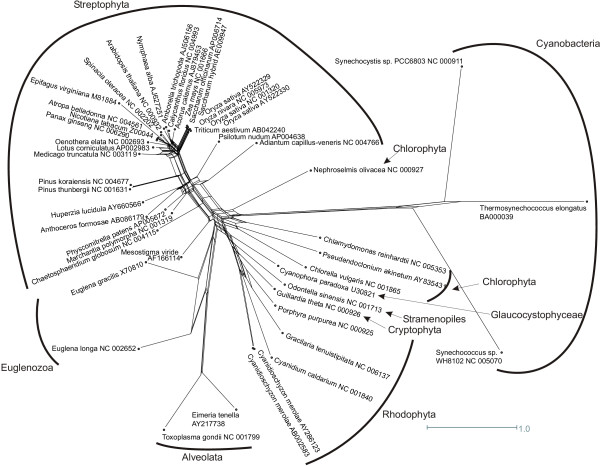
**Neighbor-net reconstruction based on GBDP distances between whole plastid genomes**. The GBDP variant used was based on equations (2) and (6) as well as BLASTN HSP search. In case of asymmetric distances, the minimum value was taken. This distance matrix achieved the globally highest c-score (0.8298), i.e., highest phylogenetic accuracy according to our reference topology, with BIONJ as tree reconstruction method. The matrix' *δ *value was 0.2013, pointing to fairly high distance quality. Bacterial outgroup taxa and major eukaryotic groups are indicated. Resolution is much higher than in best results obtained with mitochondrial genomes (Fig. 3), and most major eukaryotic groups are recovered.

**Figure 5:**
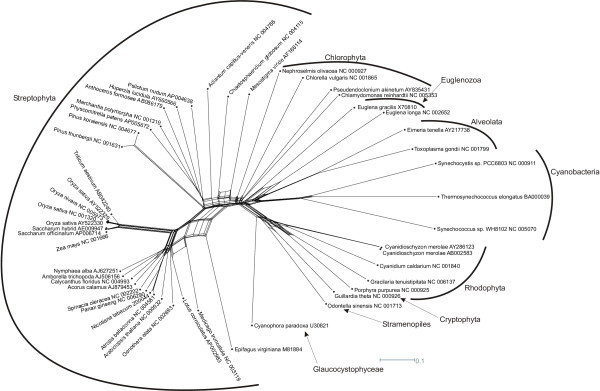
**Neighbor-net reconstruction based on GBDP distances between whole plastid genomes**. Here, the GBDP variant was derived by scaling and averaging distances based on HSP search with BLASTN and TBLASTX, respectively. Further settings were use of equations (2) and (5) and averaging of asymmetric distance values. The resulting distance matrix achieved the globally lowest *δ *value (0.1629) and, hence, globally highest distance quality. The highest c-score (topological accuracy) obtained with this matrix was 0.6596 (using BIONJ or STC as phylogenetic reconstruction method). Bacterial outgroup taxa and major eukaryotic groups are indicated. Note the apparently incorrect placement of the highly reduced genome of *Epifagus virginiana*. Other results are similar to Fig. 4; e.g., most major eukaryotic groups are well recovered.

Thus, our c-score implementation should not be affected by this potential bias, although secondary endosymbiosis events are well recovered by best GBDP trees (Figures 4, 5). For instance, both *Odontella sinensis *(stramenopiles, Bacillariophyta) and *Guillardia theta *(Cryptophyta) cluster together with red algae (Rhodophyta). The plastids of plant-like organisms within stramenopiles as well as Cryptophyta are currently assumed to be derived from red algae [43,44]. Plastids of Euglenozoa are also thought to have originated by secondary endosymbiosis, but by incorporation of algae of basal position within the "green" lineage, although exact placement of Euglenozoa plastids within that lineage is uncertain [43]. In accordance with that view, the *Euglena *genomes included in our sample show affinities to basal Streptophyta.

Interestingly, organelle ("apicoplast") genomes of Alveolata are placed by GBDP as sister taxon of Euglenozoa. These apicoplasts have only relatively recently been demonstrated to be most probably identical to plastids [58]. The "Chromalveolata hypothesis" states that Alveolata (Apicomplexa, Ciliata, and Dinoflagellates) are closely related to stramenopiles as well as to a couple of smaller groups like Cryptophyta, and that plastids found in these clades originated by a single secondary endosymbiosis event (e.g., [44]). Two recent studies [79,80] revealed that plastid-targeted GAPDH enzymes of Chromalveolata including *Toxoplasma *and *Plasmodium *(Apicomplexa) formed a distinct class unrelated to paralogous genes found in land plants, green algae, and red algae, a result which was interpreted as indicative of a single origin of plastids in Chromalveolata including Apicomplexa. On the other hand, one could ask whether genes belonging to the same family of plastid-targeted genes need to be targeted to the same kind of plastids. So far, studies based on plastid data themselves [58] indicate a sister-group relationship of Euglenozoa and Apicomplexa organelles and, hence, multiple origins of plastids in Chromalveolata, a hypothesis which is supported by GBDP applied to complete plastid genome sequences.

The position of the outgroup species is not in agreement with NCBI taxonomy, as Cyanobacteria appear as nested within Viridiplantae, the "green" plant lineage comprised of Chlorophyta (green algae) and Streptophyta (derived green algae and land plants). Within green plants, Streptophyta and Streptophytina (*Chaetosphaeridium *and land plants, i.e., *Marchantia *to *Adiantum*) are recovered. Chlorophyta is also revealed as monophyletic except for *Nephroselmis *(Chlorophyta, Prasinophyceae) which shows affinities to Streptophyta. On the other hand, Neighbor-net reveals conflicting signal located near what is expected to be the basal node of the network indicating that GBDP does not resolve the most basal branchings of plastid evolution including the origin of the green lineage. The long branch separating Cyanobacteria and plastids may be indicative of the large amount of gene loss and/or gene transfer to the nucleus which accompanied plastid creation via endosymbiosis [43].

Within land plants, positions of ferns (*Adiantum*, *Huperzia*, and *Psilotum*), mosses (*Physcomitrella*), hornworts (*Anthoceros*), and liverworts (*Marchantia*), are not resolved. Considering the problems more traditional methods of phylogenetic inference have had in resolving basal land plant relationships, this outcome should not be regarded as due to some deficiency in GBDP. The position of mosses, hornworts, and liverworts remains controversial in the literature. The most recent studies based on plastid multi-gene multiple sequence alignment indicate that these clades form a monophyletic group, but corrections for amino acid composition bias are required to achieve that result [46,81] (compare with the phylogenetic trees in, e.g. [44]).

Relationships between seed plants (*Pinus *to *Oryza*)are well recovered except for placement of *Epifagus*. *Epifagus virginiana *(Orobanchaceae) is a reduced, achlorophyllous land plant which in the course of its adaptation to parasitism on other land plants completely lost its photosynthetic ability [82]. As a consequence, *Epifagus *apparently also lost related metabolic genes, many of which would have been located within the plastid genome. Total length of its plastid genome is 70 kb, which is 44.7% and 44.9% of the plastid genome length found in *Atropa *and *Nicotiana*, respectively. As already mentioned, *Epifagus *gets misplaced at the base of Angiosperms, if GBDP is derived according to equation (2) in combination with equation (5) (Figure 5). Corrected distances (equation (3); not shown) as well as formula (2) in conjunction with formula (6) recover *Epifagus's *sister-group relationship to *Atropa *and *Nicotiana *(all in lamiids; Figure 4).

Members of the grass family (*Triticum *to *Zea*, Poaceae, monocots) seem to be misplaced near the root of the Angiosperms in both plastid/Neighbor-net reconstructions depicted. However, Figure 4 also shows alternative splits indicating a sister-group relationship between *Acorus calamus *and Poaceae in accordance with monocot monophyly. The position of the monocot taxa remained controversial in analyses of multiple plastid gene alignments from a small number of selected flowering plant taxa [46]. Agreement with studies including several hundreds of species and few genes (e.g., Savolainen et al. [83]) was only recently achieved by including a more representative set of taxa from incompletely sequence plastid genomes [47]. As with respect to basal land plant resolution, we conclude that these results point to problems inherent to plastid data (or due to insufficient taxon sampling). We see no evidence that GBDP performs less well than do more commonly used approaches based on multiple sequence alignment.

## Conclusion

As a contribution to the growing field of phylogenomics, we here inferred GBDP phylogenies from completely sequenced plastid and mitochondrial genomes. In search for an optimal approach, a number of modifications of GBDP were examined. The effects of the individual distance function parameters on distance matrix quality and on the accuracy of the resulting trees were examined by multiple linear regression. Such a framework can also be used in future studies to assess other phylogenetic distance methods.

It turned out that accuracy of the resulting trees is in good accordance with the treelikeness of the underlying distance matrices as measured by their *δ *value. As a valuable approach in general, *δ *values should be particularly useful in future research if a choice has to be made between different distance methods but a reference tree is unavailable or cannot be used because additional independent phylogenetic evidence is aimed at. For instance, it would be interesting to compare *δ *values obtained with GBDP with those obtained with other methods which infer distances directly from whole genomes (e.g., [5-12,19,20]). However, *δ *values may also be used to find the optimal substitution model for aligned nucleotide or amino acid sequences [14,52]. If trees are to be inferred using distance methods anyway, this approach may be more consistent than the more commonly used likelihood-based criteria [74,84,85]. If unambiguous multiple sequence alignment is difficult, it may also be useful to infer distances matrices directly from single loci (e.g., [34-36]). *δ *values could be used to compare these distance methods with each other and with distance derived from different alignments of the same sequence data. The *δ *approach is not restricted to sequence data but could also be used to assess other kinds of phylogenetic distance functions such as those for restriction endonuclease or immunological data [14] or even distance formulae based on the distribution of parasite species on their hosts (e.g., [86]). Note that the Q criterion as formulated by Guindon and Gascuel [54] is computed in almost the same way as *δ*; it would be interesting to compare these two quartet-based measures of treelikeness in future studies.

Regarding the GBDP variants, it became obvious that distances based on breakpoints are of little use. On the other hand, distances based on the proportion of "matched" HSP length to average genome length performed best in tree estimation. Furthermore, replacing BLASTN by TBLASTX in the GBDP approach led to a small increase in accuracy. Combining TBLASTX and BLASTN HSP search may also be beneficial. Interestingly, combination works almost equally well before and after distance computation. We showed that other factors do not have much effect on the resulting phylogenies. Distance quality also had a much larger effect than reconstruction methods. If applied to high quality distance matrices, all agglomerative methods we examined performed well except for UPGMA. In accordance with earlier studies, Neighbor-net was found to be particularly useful to visualize the results. It provides an efficient way to demonstrate the tree-likeness of the distance matrices and the amount of conflicting signal or unambiguous support for certain phylogenetic relationships in the data.

With respect to the phylogenetic outcome, we conclude that completely sequenced mitochondrial genomes suffer from saturation. Application of GBDP to mitochondrial genomes may be more valuable within major eukaryotic lineages such as Metazoa. In contrast, plastid GBDP led to much more accurate phylogenies if based on the most treelike distance matrices. Besides recovering major plant lineages as recognised in current NCBI taxonomy, best plastid GBDP distances reveal a sister-group relationship between Apicomplexa and Euglenozoa plastids, indicating the plastid evolution in Chromalveolata may have been more complicated than currently recognised.

## Authors' contributions

Programming was done by AFA, SRH and BRH. MG conceptualised the study and wrote the manuscript. All authors read and approved the final manuscript.

## Supplementary Material

Additional File 1 Tab delimited text file containing the characteristic parameters and the quality measures of the distance matrices used for regression analyses, as well as all trees inferred in the course of this study in Newick format. Click here for file

## Figures and Tables

**Table 1:** Regression analysis. Results of step-wise multiple linear regression based on the AIC criterion. Left side: c-score in dependence on all qualitative variables: equation (1), (2), (3), or (4); equation (5) or (6); average, minimum, or maximum of asymmetric distance values; BLASTN, TBLASTX, BLASTN after TBLASTX, or matrix combination; UPGMA, NJ, BIONJ, FastME, or STC. Right side: *δ *value in dependence on all variables. Only those explanatory variables judged as significant in at least one of the two approaches are included; those not shown were removed in the course of the step-wise procedures and did not contribute to the final regression model.

Regression analysis								
	**cscore **(adjusted *R*^2 ^= 0.775)				*δ ***value **(adjusted *R*^2 ^= 0.888)			

explanatory var.	coefficient	standard error	*t *value	*P*(*x *> |*t*|)	coefficient	standard error	*t *value	*P*(*x *> |*t*|)

(Intercept)	0.032509	0.010895	2.984	0.00292	0.533266	0.008668	61.522	< 2^-16^
Plastids	0.123627	0.006290	19.655	< 2^-16^	-0.172317	0.005779	-29.820	< 2^-16^
BLASTN + TBLASTX	0.047477	0.008895	5.337	1.18^-07^	-0.044311	0.008172	-5.422	1.84^-07^
Matrix Averaging	0.024675	0.008895	2.774	0.00565	not significant			
TBLASTX	0.044962	0.008895	5.055	5.18^-07^	-0.041812	0.008172	-5.116	7.82^-07^
Eq. (6)	not significant				0.043077	0.005779	7.455	3.48^-12^
Eq. (2)	0.437622	0.008895	49.197	< 2^-16^	-0.184422	0.008172	-22.567	< 2^-16^
Eq. (3)	0.380340	0.008895	42.757	< 2^-16^	-0.125118	0.008172	-15.310	< 2^-16^
Eq. (4)	0.247183	0.008895	27.788	< 2^-16^	-0.088696	0.008172	-10.853	< 2^-16^
STC	-0.040611	0.009945	-4.083	4.81^-05^				
UPGMA	-0.029744	0.009945	-2.991	0.00285				
